# Accuracy of next-generation sequencing for molecular profiling of small specimen of lung cancer: a prospective pilot study of side-by-side comparison

**DOI:** 10.1186/s13000-022-01255-y

**Published:** 2022-10-12

**Authors:** Xiaosong Ben, Dan Tian, Weitao Zhuang, Rixin Chen, Sichao Wang, Zihao Zhou, Cheng Deng, Ruiqing Shi, Songlin Liu, Dongkun Zhang, Jiming Tang, Liang Xie, Haiyu Zhou, Zhou Zhang, Min Li, Xuanye Zhang, Guibin Qiao

**Affiliations:** 1 Department of Thoracic Surgery, Guangdong Provincial People’s Hospital, Guangdong Academy of Medical Sciences, 106 Zhongshan Second Road, Guangzhou, 510080 China; 2 Department of Medical Oncology, State Key Laboratory of Oncology in South China, Collaborative Innovation Center for Cancer Medicine, Sun Yat-sen University Cancer Center, 510060 Guangzhou, China; 3 Research Center of Medical Sciences, Guangdong Provincial People’s Hospital, Guangdong Academy of Medical Sciences, Guangzhou, China; 4 Department of Data Science, Burning Rock Biotech, Guangzhou, China; 5 Department of Medicine, Burning Rock Biotech, Guangzhou, China

**Keywords:** Non-small cell lung cancer, Next generation sequencing, Molecular profiling, Small specimen, Core-needle biopsy

## Abstract

**Background:**

Successful practice of precision medicine in advanced lung cancers relies on therapeutic regimens tailored to individual molecular characteristics. The aim of this study was to investigate the accuracy of small specimens for molecular profiling using next-generation sequencing (NGS).

**Methods:**

Genetic alternations, tumor mutational burden (TMB), status of microsatellite instability (MSI), and expression of programmed death ligand 1 (PD-L1) were compared side-by-side between the concurrently obtained core needle biopsy (CNB) and resection specimens in 17 patients with resectable non-small cell lung cancers.

**Results:**

DNA yield and library complexity were significantly lower in CNB specimens (both *p* < 0.01), whereas the insert size, sequencing depth, and Q30 ratio were similar between the matched specimens (all *p* > 0.05). The total numbers of genetic alternations detected in resection and CNB specimens were 186 and 211, respectively, with 156 alternations in common, yielding a specific concordance rate of 83.9%. The prevalence of mutations in 8 major driver genes was 100% identical between surgical and CNB specimens, though the allele frequency was lower in CNB specimens, with a median underestimation of 57%. Results of TMB were similar (*p* = 0.547) and MSI status was 100% matched in all paired specimens.

**Conclusions:**

Pulmonary CNB specimens were suitable for NGS given the satisfactory accuracy when compared to corresponding surgical specimens. NGS results yielding from CNB specimens should be deemed reliable to provide instructive information for the treatment of advanced lung cancers.

**Supplementary Information:**

The online version contains supplementary material available at 10.1186/s13000-022-01255-y.

## Background

The continuing exploration of the molecular landscape and rapid development of pharmaceutical products have been opening new frontiers for the systemic therapies of advanced lung cancers. With the development of multimodal treatment, each patient can be tagged with numerous molecular characteristics to assist in regimen prescription and prediction of treatment response. These characteristics include but not limited to the specific gene mutations, tumor mutational burden (TMB), status of microsatellite instability (MSI), and expression of programmed death ligand 1 (PD-L1) [1, 2]. Genetic testing, which is one of the tools of molecular profiling, has been recommended by clinical guideline to guide the first-line treatment for advanced lung cancer. For patients with unresectable or metastatic cancer, genetic and other molecular testing is usually performed on relatively small core needle biopsy (CNB) or fine needle aspiration (FNA) samples. With the discovery of increasing number of targetable genomic aberrations, the traditional method of sequential single-gene testing is limited by the small biopsy sample [3, 4], and is being substituted by next-generation sequencing (NGS). The lower requirement for DNA amount and high throughput of NGS hold great promise to provide comprehensive molecular analysis for inoperable patients [5].

Adequacy and integrity of tumor tissues remain a major obstacle to successful clinical NGS testing [6]. Recently, the feasibility of using small specimen (e.g. CNB or FNA) for NGS had been validated in many studies [6–8]. However, its accuracy was rarely investigated due to the difficulty in accessing the concurrently acquired biopsy and the corresponding resection specimens. As a result, it remains unclear that to what extent a biopsy sample can represent the actual molecular status of the malignancy, given the temporal and spatial heterogeneity of lung cancer [9]. Several studies have addressed the adequacy of small biopsy samples for NGS [8, 10–13] , yet to the best of our knowledge, most of these studies were retrospective in nature, and did not provide further knowledge about the accuracy of molecular testing using small tissue specimens.

In the current study, we first performed a prospective side-by-side comparison between the concurrently acquired core needle and surgical specimens in the patients with resectable lung cancer. We aimed to provide more evidence for using molecular profiling of biopsy sample to guide the systemic treatment in those who are medically inoperable, or with advanced pulmonary malignancies.

## Methods

### Patients

A total of 17 patients with non-small cell lung cancer from Guangdong Provincial People’s Hospital were enrolled in this prospective pilot study from January 7^th^ to October 28^th^, 2019. Demographic and clinicopathologic information including age, sex, smoking status, surgical approach, tumor size, location and pathological reports were collected from electronic medical record (EMR), and desensitized when performing statistical analysis. All patients were clinically diagnosed with resectable pulmonary neoplasms. Preoperative workup included thoracic and abdominal computed tomography (contrast-enhanced), cranial magnetic resonance imaging, whole-body bone scan or positron emission tomography-computed tomography (PET-CT) alone. Pathological stage was assessed based on the eighth edition of AJCC TNM staging system.

### Sample acquisition

**Fig. 1:**
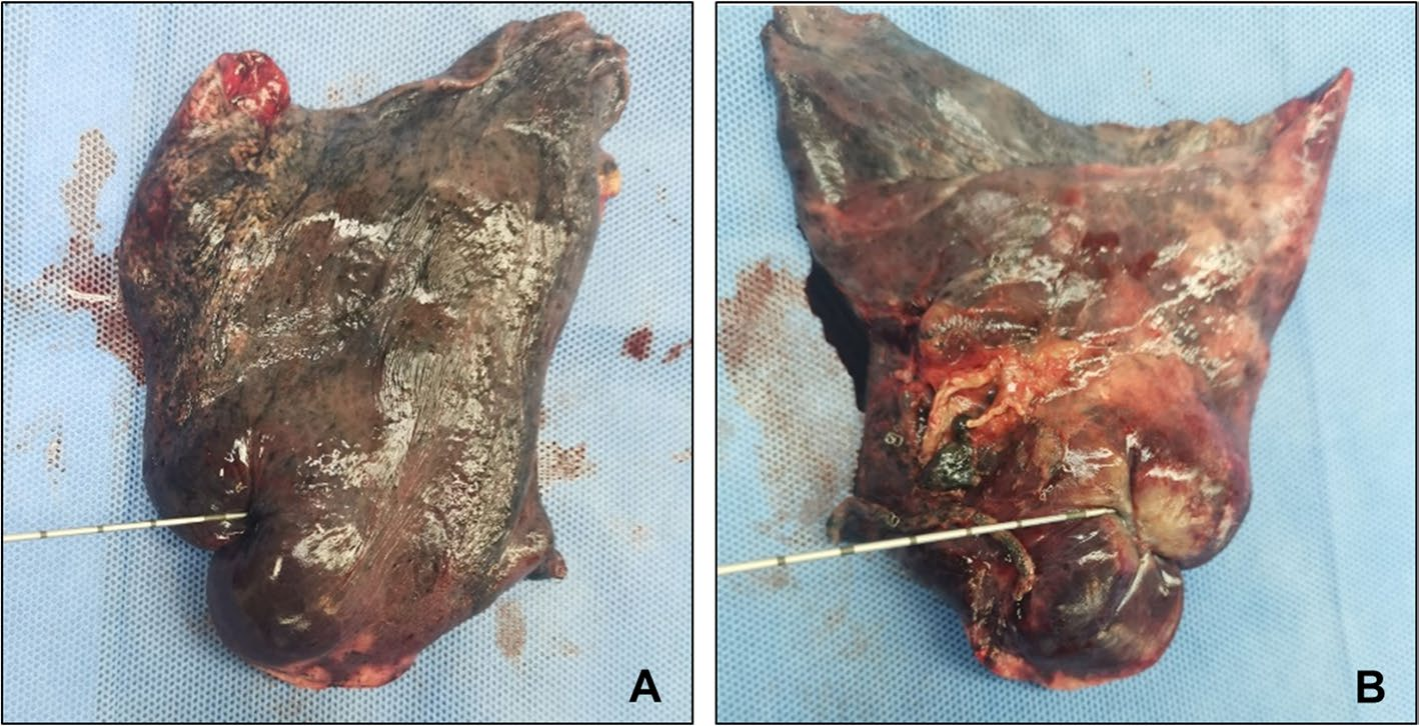
Representative picture of intraoperative core needle biopsy. **A** Needle puncture performed at the point of maximum diameter of the lesion. **B** Resection of the lesion after biopsy to confirm an appropriate puncture

For practical reasons and the best interest of the patients, all core needle biopsy samples were obtained intraoperatively to avoid any potential risk of tumor dissemination along needle passage. An 18-gauge Max-Core™ disposable core needle with 22-mm penetration depth (BARD Biopsy, AZrizona, USA) was used for specimen punctuation in all patients. One pass to the lesion on the level with maximum diameter was obtained immediately after the removal of lung tissue from thoracic cage (Fig. 1). All samples were immediately fixed in 10% neutral buffered formalin solution and embedded in paraffin (FFPE) between 6 and 24 hours [14]. White blood cells were collected and used for filtering germline mutations. All samples were sent to a Clinical Laboratory Improvement Amendments (CLIA)-certified laboratory (Burning Rock Biotech, Guangzhou, China) for genetic sequencing and IHC staining.

### Molecular and pathological testing

A total of 10 paraffin sections were required for both biopsy and resection specimens for NGS. DNA was extracted using the QIAamp DNA FFPE tissue kit (Qiagen, Waltham, MA, USA) and QIAamp Circulating Nucleic Acid kit (Qiagen, Waltham, MA, USA) according to the manufacturer’s instructions. The concentration of DNA was measured by the Qubit dsDNA assay (Life Technologies, Waltham, MAassachusetts, USA). A total of 50-200 ng DNA were performed for further targeted sequencing. DNA fragmentation was performed using Covaris M220 (Covaris Inc., Woburn, MA, USA), followed by end repair, phosphorylationphosphorylation, and adaptor ligation. Fragments of size 200 – 400bp were selected by AMPure bead (Agencourt AMPure XP Kit, Beckman Coulter Inc., Brea CA, USA), followed by hybridization with capture probes baits, hybrid selection with magnetic beads and PCR amplification. The OncoScreenPlus panel (Burning Rock Biotech, Guangzhou, China) which contain 520 cancer-related genes, including all targets of targeted therapies, spanning 1.86Mb of human genome was used [15]. Among them, whole exons of 310 genes and critical exons, introns, and promoter regions of the remaining 210 genes were captured. A high-sensitivity DNA assay was then performed using Bioanalyzer (Agilent 2100, Agilent Technologies Inc., Santa Clara, CA, USA) to assess the quality and size of the fragments and indexed samples were sequenced on Nextseq500 sequencer (Illumina, Inc., San Diego, CA, USA) with pair-end reads. Additionally, the length distribution of 63 microsatellite loci was evaluated using NGS to determine the MSI status.

Sequencing data were mapped to the human genome (hg19) using BWA aligner 0.7.10. Local alignment optimization, variant calling and annotation were performed using GATK 3.2, MuTect uTect (both from Broad Institute, Cambridge, MA, USA), and VarScan (Genome Institute, Washington University, Washington D.C, USA). Loci depth less than 100 were filtered out. InDels and SNVs calling required at least five and eight supporting reads. Variant with population frequency higher than 0.1% according to the ExAC, 1 000 Genomes, dbSNP, ESP6500SI-V2 database were grouped as SNP and excluded from further analysis. Remaining variants were annotated with ANNOVAR and SnpEff v3.6 software. DNA translocation analysis was performed using both Tophat2 and Factera 1.4.3. Statistical analysis was performed using the SPSS version 23.0 (SPSS Inc., Chicago, IL, USA) and MedCalc Statistical Software version 19.6 (MedCalc Software Ltd., Ostend, Belgium). Copy number variations were analyzed based on the depth of coverage data of capture intervals. The average coverage of all captured regions was used to normalize the coverage of different samples to comparable scales. Copy number was calculated based on the ratio between the depth of coverage in tumor samples and average coverage of an adequate number of samples without copy number variations as references per capture interval. Copy number variation is called if the coverage data of the gene region was quantitatively and statistically significant from its reference control. DNA fusions were analyzed as previously described [16].

In this study, four parameters were used for the quality control of genetic sequencing, including library complexity, insert size, median depth, and Q30 ratio. The library complexity reflects the sample size of all input samples finally included in the library and sequenced. Insert size is used for the assessment of DNA degradation, where a lower value indicates a higher degradation of DNA. Q30 ratio is the proportion of reads that have a sequencing accuracy of more than 99.9%, which reflects the quality of genetic sequencing. In this work, the criterion used for library complexity, insert size, median depth, and Q30 ratio was ≥ 20%, ≥ 150 bp, 500×, and ≥ 80%, respectively. Specific rate of concordance is defined as the number of shared genetic alternations over the number of genetic alternations detected in surgical specimens.

IHC analysis was conducted on another consecutive paraffin section from both biopsy and resection specimen. All samples were pretreated and stained with the PD−L1 antibody 22C3 mouse monoclonal primary antibody (Agilent Technologies Inc., Santa Clara, CA, USA) in accordance with the manufacturer’s instruction. Tumor proportion score (TPS), which is the ratio of stained cancer cells over all viable cancer cells, was used for measurement of PD-L1 expression.

### Statistical analysis

Clinicopathological data from EMR was presented as frequency, mean, or median as appropriate. Data of quality control was tested using Wilcoxon rank sum test. TMB between resection and biopsy specimens was compared using related-samples Wilcoxon signed rank test. Concordance was assessed by Cohen’s kappa (κ) coefficient of agreement. The level of concordance was classified as poor (κ < 0.00), slight (κ = 0.00-0.20), fair (κ = 0.21-0.40), moderate (κ = 0.41-0.60), substantial (κ= 0.61-0.80), and almost perfect (κ = 0.81-1.00) (12). A two-sided *p* value of <0.05 was considered statistically significant.

## Results

### Clinicopathological characteristics

The baseline characteristics of the study cohort are summarized in Table 1. A total of 17 patients were enrolled in this study, including 10 males and 7 females, with a mean age of 63.7 years old. There were 4 current smokers, 4 ever-smokers, and 9 non-smokers. The median tumor size was 20 mm, with the minimal one being 5 mm and the maximal one being 80 mm. All patients underwent surgical resection, including 12 cases of lobectomy, one case of segmentectomy, and four cases of wedge resection. There were 15 cases of adenocarcinoma, 1 case of squamous cell carcinoma (P05), and 1 case of large cell carcinoma (P07). The majority of adenocarcinomas were acinar predominant (12 out of 15). Papillary pattern (P04), solid growth pattern (P11), and neuroendocrine differentiation (P03) were found in the other three patients, respectively. No lymphovascular invasion was reported in all these tumors. However, two patients were found to have visceral pleura invasion (P12&17). The majority of patients had early-stage disease, with 12 patients at stage I and one patient at stage II. The other 4 patients had stage III lung cancers, either contributed by lymph node metastasis (3 out of 4 cases, P04, 15&17) or large tumor size (1 out of 4 cases, P11). The median percentage of tumor cells in surgery samples was 50% with a range of 5-80%, which was 60% with a range of 10-80% in biopsy samples. Moreover, the percentages of tumor cells in surgery and biopsy samples were comparable (*p* > 0.05).

**Table 1:** Clinicopathological characteristics of enrolled patients

**Clinicopathological characteristics**	***n*=17**
**Age, years**	
Mean (range)	63.7 (44-86)
**Sex**	
Male/Female	10/7
**Smoking status**	
Current/Ever/Never	4/4/9
**Tumor size, mm**	
Median (range)	20 (5-80)
**Tumor location**	
RUL/RML/RLL	8/1/4
LUL/LLL	3/1
**Surgical approach**	
Wedge/Segmental/Lobal	4/1/12
**Histology**	
Adenocarcinoma	15
Squamous cell carcinoma	1
Large cell carcinoma	1
**Predominant histologic pattern**	
Acinar/papillary/solid/others	12/1/1/3
**Lymph node metastasis**	
N0/N1/N2/N3	13/1/3/0
**Pathological TNM stage**	
I/II/III/IV	12/1/4/0

*RUL* Right upper lobe, *RML* Right middle lobe, *RLL* Right lower lobe, *LUL* Left upper lobe, *LLL* Left lower lobe

### Quality control of DNA sequencing

**Fig. 2:**
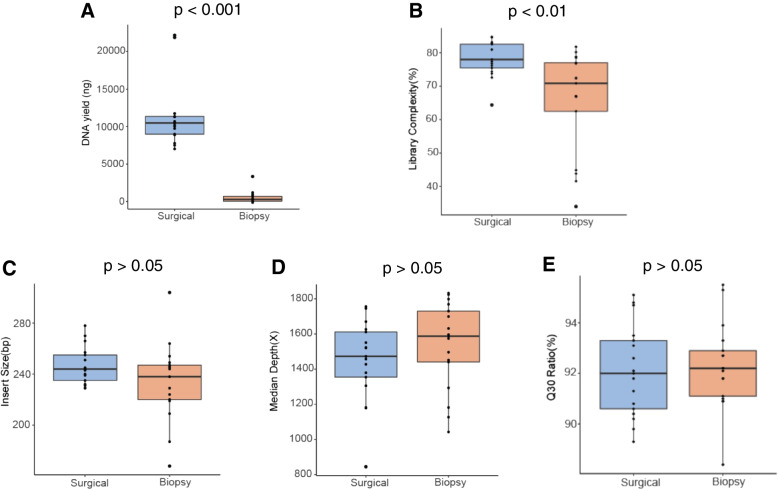
Quality control measures of targeted next generation sequencing for biopsy and surgery. **A** DNA yield. **B** Library complexity. **C** Insert size. **D** Median depth. **E** Q30 ratio

The adequacy and integrity of specimens are the premise of successful and accurate sequencing. To ensure the quality of specimens, a process of quality control was compulsory before any further testing. First of all, we quantified the DNA yield from each specimen, and the result showed a significant less DNA yield in the CNB samples than the amount obtained from the resection samples (Table 2, Fig. 2A, *p* < 0.0001). Similarly, the library complexity was significantly higher in resection specimens than that in biopsy specimens (Table 2, Fig. 2B, *p* < 0.01). The quality of genetic sequencing was subsequently evaluated by insert size (bp), sequencing depth (×), and Q30 ratio (%). The CNB and resection specimens were found to have no difference in regards of these three parameters (Table 2, Fig. 2C, D, and E, with all *p* > 0.05). In short, the lower yield in biopsy specimens did not impair the quality of DNA for sequencing. The qualification data of extracted DNA of each sample are described in Table S1.

**Table 2:** Quality control for biopsy and resection specimens.

	**Biopsy**	**Resection**	***P*-value**^a^
**Library complexity**, median (%)	70.9	78.0	< 0.01
**Insert size**, median (bp)	238	244	0.1429
**Median depth**, median ($\times$)	1587	1472	0.2485
**Q30 ratio,** median (%)	92.2	92.0	0.5579
**DNA yield,** median (ng)	329	12005	< 0.001

*bp* Base pair; ^a^Wilcoxon rank-sum test

### Concordance of mutational status

**Fig. 3:**
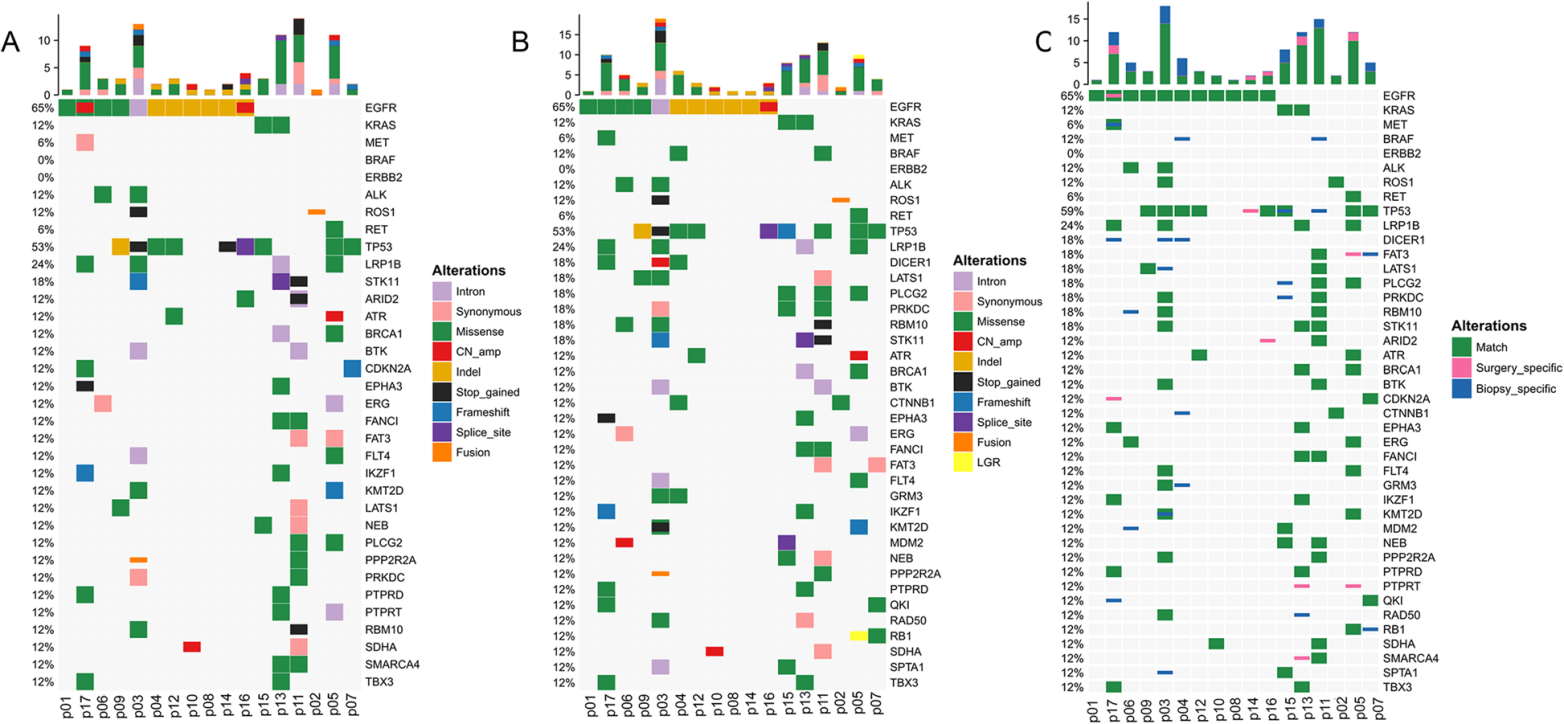
Mutational landscape and concordance analysis. **A** Genetic profiles of the surgical resection specimen; **B** Genetic profiles of the CNB specimen; **C** The comparison of genetic profiles of resection specimen and matched CNB sample. Green indicates mutations detected from both sources, pink indicates mutations that were present only in the surgical resection specimens, and blue indicates mutations that were present only in the core needle biopsy samples. CNB: core needle biopsy specimens; CN_amp: copy number amplification; InDel: small insertion and deletion; LGR: large genomic rearrangement

The mutational landscape of all resection and CNB samples is depicted in Fig. 3A and B, respectively. A total of 186 genetic alterations were detected in the resection specimens from 17 patients, which mainly included 87 (46.8%) missense mutations, 22 (11.8%) copy number amplifications, and 21 (11.3%) synonymous mutations. In contrast, a total of 211 genetic alterations were detected in the CNB specimens, which primarily constitute of 97 (46.0%) missense mutations, 36 (17.1%) copy number amplifications, and 23 (10.9%) synonymous mutations (Supplementary Fig. S1). Comparative analysis of these alterations revealed 156 alternations in common. In other words, 30 out of 186 and 55 out of 211 alternations were specific to the resection and CNB specimens, respectively. Therefore, the specific concordance rate of NGS using CNB specimen with regard to resection specimen was 83.9% (156/186) (Fig. 3C).

The sequencing depth of the specific alternations was comparable with that of the matched alterations (*p* > 0.05, Supplementary Fig. 2A). A further comparative analysis of the allele frequency (AF) revealed that the specific alternations had a significantly lower AF than the matched alternations (*p* < 0.01, Supplementary Fig. S2). Among the detected alternations, the eight-major driver mutations of lung cancer (*EGFR*, *ALK*, *ERBB2*, *MET*, *RET*, *ROS1*, *BRAF,* and *KRAS*) could be found in 14 out of 17 (82.4%) patients using both surgical and CNB specimens (Table 3). The remaining 3 patients harbored no eight-major driver mutations both in surgical and CNB specimens (Table 3). The concordance of eight-major driver mutations between CNB and surgical samples reached 100%. In particular, the allele frequency of the predominant mutation, *EGFR*, was obviously higher in surgical specimens in 6 out of 10 patients, and approximated to each other among another 3 patients (Table 3). A median underestimation of 57% allele frequency was found in CNB specimens.

**Table 3:** Details of overall genetic alternations and mutations of 8-major driver genes detected in biopsy and resection specimens

**Patient Number**	**Tumor size(mm)**	**Surgery**					**Biopsy**				
		**Total No. of alternations**	**Percentage of tumor cells (%)**	**Gene**	**Description**	**AF (%)**	**Total No. of alternations**	**Percentage of tumor cells (%)**	**Gene**	**Description**	**AF (%)**
P01	20	3	80	*EGFR*	p.Leu858Arg	18.96	4	60	*EGFR*	p.Leu858Arg	13.31
				*EGFR*	p.Thr790Met	17.79			*EGFR*	p.Thr790Met	18.57
P02	15	3	50	*ROS1*	CD74-ROS1	23.67	3	30	*ROS1*	CD74-ROS1	21.32
P03	30	33	40	*ALK*	p.Pro719His	12.92	44	70	*ALK*	p.Pro719His	16.67
P04	15	3	60	*EGFR*	p.Glu746_Ala750del	20.75	13	70	*EGFR*	p.Glu746_Ala750del	20.88
P05	5	30	80	*/*			28	80	*/*		
P06	22	4	70	*EGFR*	p.Leu858Arg	19.15	8	70	*EGFR*	p.Leu858Arg	25.74
				*ALK*	p.Pro542Arg	13.42			*ALK*	p.Pro542Arg	5.06
P07	17	7	10	*/*			10	10	*/*		
P08	15	3	20	*EGFR*	p.Glu746_Ala750del	14.22	1	50	*EGFR*	p.Glu746_Ala750del	8.16
P09	25	3	40	*EGFR*	p.Leu858Arg	21.06	4	60	*EGFR*	p.Leu858Arg	12.73
P10	20	6	50	*EGFR*	p.Glu746_Ala750del	10.58	5	70	*EGFR*	p.Glu746_Ala750del	7.97
P11	80	33	30	*/*			36	60	*/*		
P12	11	6	50	*EGFR*	p.Leu747_Pro753delinsSer	8.5	5	60	*EGFR*	p.Leu747_Pro753delinsSer	8.16
P13	20	18	50	*KRAS*	p.Gly12Cys	15.52	14	60	*KRAS*	p.Gly12Cys	26.59
P14	12	4	5	*EGFR*	p.Leu747_Thr751del	16.49	1	50	*EGFR*	p.Leu747_Thr751del	4.57
P15	25	9	70	*KRAS*	p.Gly12Val	8.13	14	50	*KRAS*	p.Gly12Val	8.45
P16	21	5	60	*EGFR*	p.Pro772_His773dup	36.71	3	80	*EGFR*	p.Pro772_His773dup	36.44
P17	30	16	70	*EGFR*	p.Leu858Arg	23.4	21	50	*EGFR*	p.Leu858Arg	15.67

*AF* Allele frequency, *No.* Number, *ALK* Anaplastic lymphoma kinase, *EGFR* Epidermal growth factor receptor, *KRAS* Kirsten rat sarcoma viral oncogene, *ROS-1* ROS proto-oncogene, *del* Deletion, *dup* Duplication, *delins* Deletion and insertion

**Fig. 4:**
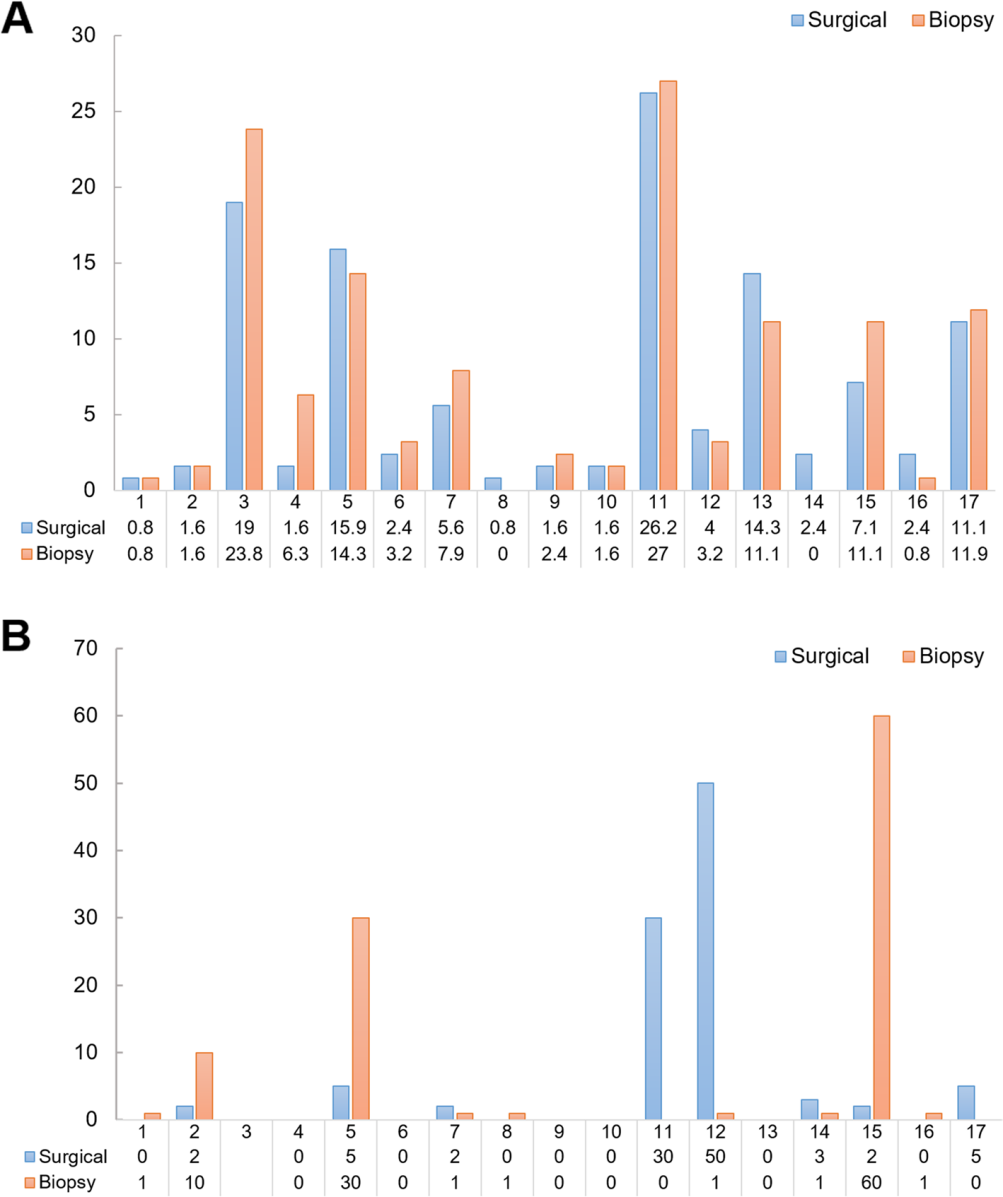
**A** Tumor mutational burden (in Mutation#/Mb) of paired specimens; **B** PD−L1 expression (in TPS) paired specimens. *Specimens from patient 03 did not satisfy the standard of quality control. PD-L1, programmed death ligand 1; TPS, tumor proportion score

TMB of resection and matched CNB specimens was then calculated as shown in Fig. 4A. The average resection specimen-based TMB was 6.97 mutations/Mb (ranging from 0.8-26.3) and the average CNB-based TMB was 7.47 mutations/Mb (ranging from 0 to 27) (Fig. 4A, B). We also found resection specimen-based TMB and CNB-based TMB was comparable (*p* = 0.547, Supplementary Fig. S3A). Next, the correlation between CNB-based TMB and resection specimen-based TMB was investigated. CNB-based TMB positively correlated with resection specimen-based TMB (Pearson *r* = 0.959, *p* < 0.0001, Supplementary Fig. 3B). Additionally, the status of MSI was totally matched in all paired samples.

### Concordance of PD-L1 expression

The expression of PD-L1 is classified into three grades in our study: negative (TPS < 1%), low expression (TPS 1−49%), high expression (TPS ≥ 50%). Patient 03 failed to provide adequate cancer tissue for IHC staining of PD−L1.The details of PD-L1 expression of each patient are depicted in Fig. 4B, with an overall agreement proportion of 56.3% (9 out of 16 cases). The agreement of the ordered categorical variables was assessed using Cohen’s quadratic weighted κ coefficient of agreement. The concordance of PD-L1 expression between paired biopsy and surgical specimens was not satisfactory, with a κ value of 0.403 (95% CI, 0.090−0.718).

### The clinical outcomes of patients

A total of 12 of 17 patients did not receive postoperative adjuvant therapy. All these 12 patients were at stage I. At the time of manuscript revision (August 2022), these 12 patients were still free of tumor recurrence with a median recurrence-free survival (RFS) of 40 months (range: 35.6-44.3 months). The adjuvant therapy information of P11 who had stage IIIA lung adenocarcinoma (LUAD) was unavailable. The remaining 4 patients (P04, P05, P15, and P17) received different adjuvant therapy regimens. P04 (a 60 years female never-smoker) with stage IIIA LUAD who harbored a *EGFR* exon 19 deletion p.Glu746_Ala750del received gefitinib as adjuvant therapy. The patient was still free of tumor recurrence in August 2022 with an RFS of 44 months and counting. P05 (a 61 years male ever-smoker) with stage IIB lung squamous cell carcinoma (LUSC) who harbored no actionable alterations but had PD-L1 expression (TPS: 5% in the surgical sample and 30% in the CNB sample) received platinum plus paclitaxel and a programmed cell death protein 1 (PD-1) inhibitor pembrolizumab as adjuvant therapy. She was still free of tumor recurrence in August 2022 with an RFS of 39.7 months and counting. P15 (a 59 years male current smoker) with stage IIIA LUAD and *KRAS* p.Gly12Cys received platinum plus paclitaxel as adjuvant therapy. He was still free of tumor recurrence in August 2022 with an RFS of 37 months and counting. P17 (a 51 years male current smoker) with stage IIIA LUAD harboring *EGFR* p.Leu858Arg received platinum plus paclitaxel and radiotherapy as adjuvant therapy. The patient underwent a tumor recurrent with an RFS of 21 months.

## Discussion

NGS has been known by its capability to provide comprehensive mutational profiling using as low as 10 ng DNA extracted from small biopsy specimens [17]. This technical revolution is getting more accessible and affordable in the era of precision medicine. A successful practice of targeted therapy and immunotherapy usually requires a comprehensive characterization of molecular landscape of each individual patient. First-line treatment in advanced lung cancer patients usually relies on testing of small biopsy sample only, posing a considerable challenge in the overall coordination of sample acquisition, storage, processing, and the testing technique itself. To the best of our knowledge, this is the first prospective pilot study to evaluate the agreement of NGS results in matched resection and biopsy specimens of lung cancer.

We prospectively enrolled 17 patients without knowledge of their histology or exact pathological stage before performing the NGS. For practical reason, only patients with resectable non-small cell lung cancer were selected in order to concurrently obtain the corresponding surgical specimens. Previous studies reported an acceptable unsuccessful rate of NGS using small biopsy samples, ranging from 2.7% to 20% [8, 10–13]. Zheng et al. performed NGS analysis on 131 biopsy and 110 FNA FFPE samples of lung cancers, with 3.8% and 2.7% unsuccessful tests due to insufficient tumor tissues [13]. A smaller study successfully yield full NGS reports in 16 out of 17 (94%) small biopsy samples using a 467-gene panel [10]. In the current study, all samples survived the process of quality control. NGS was successfully performed in all 17 patients, which might be contributed by our careful intraoperative selection of the biopsy site followed by a standardized protocol of storage, transport, and laboratory processing. This design was critical because 11 out of 17 patients had their largest tumor diameter shorter than the penetration length (22 mm) of the core needle. Many controllable factors in the processing procedure can be modified to increase the success rate of performing NGS on small samples. For example, Padmanabhan et al. had their success rate improved from 68% to 94% after implementation of rapid on-site evaluation and reduction of FFPE block facing to only once [7].

Adequacy is no longer the bottleneck for performing NGS in small biopsy tissues, whereas its accuracy was rarely investigated and remained a major question to be answered. We have shown a relatively high concordance of genetic alternations between matched surgical and biopsy specimens in the current study, and identical common driver mutations were found in 100% patients (14 in total) through the CNB samples. Although the allele frequency was relatively lower in CNB samples, with a median underestimation of 57%, its NGS results had provided us with sufficient information for the initiation of systemic treatment. It had been reported that allele frequency can be a potential predictive factor of TKI treatment efficacy in patients with *EGFR*L858R mutation. In this regard, the NGS result of CNB sample should not be applied to treatment efficacy prediction. Additionally, it seemed that no correlation existed between tumor size and allele frequency of mutations, as well as the number of genetic alternations in our study. A larger sample size is required to answer this question. Interestingly, more genetic alternations was detected in the CNB than resection samples (211 vs. 186), including two important driver mutations of *BRAF* (p.Glu549Gln in P04 and p.Ala762Val in P11). The incomplete snapshot of mutational status captured by either tissues could be inherently contributed by the intratumoral heterogeneity [18]. Theoretically speaking, needle puncture at the largest diameter could include as more cellular clones as possible. In contrast, the consecutive paraffin sections of surgical sample might only represent one part of the lesion. As expected, the allele frequency of these specific alternations were significantly lower than the matched alternations, indicating the scarcity of those clones (Supplementary Fig. S2B). These results supported the reliability to use CNB specimens as a surrogate to provide instructive mutational information for targeted therapy, except for its traditional use for diagnostic purpose.

Recently, some authors had evaluated the agreement of targeted NGS results between liquid biopsy and their tissue counterpart in patients with advanced lung cancer. Although less invasive than tissue biopsy, the results of blood-based liquid biopsy were not ideal enough at the current stage, with a rate of concordance being 53.3% to 67.8% depending on specific genes [19]. In contrast, researchers found a surprising high concordance of mutation profile between centrifuged supernatant from small biopsy specimens and their corresponding tissue samples [20, 21]. This discovery confirmed the representativeness of the small biopsy samples from another aspect, and helps to avoid repeat biopsies when the specimen is insufficient for testing [20, 21].

As immunotherapy becoming the first-line treatment for advanced lung cancer, potential predictive biomarkers of immune checkpoint inhibitor (ICI), such as PD−L1, TMB and MSI, were also compared between biopsy and resection specimens in this study. The expression of PD−L1 had an undesirable concordance rate of 56.3% and a κ value of 0.403 (Fig. 4B) in trivariate categorization (negative, low expression, high expression), which might be caused by the limited amount of tissue after its use for NGS. This suggests us that immunohistochemistry (IHC) staining of PD−L1 should be performed on another independent core sample. Kitazono et al. retrospectively evaluated the expression of PD−L1 in 79 matched small biopsy and resected specimens, yielding a concordance rate of 92.4% and κ value of 0.8366 [22]. Gradecki et al. reported similar results in 51 paired samples, with a concordance rate of 92.2% and κ value of 0.70 (95% CI, 0.43−0.98) [23]. However, heterogeneous expression of PD−L1 was observed and this can be overcome by optimizing the number of cores obtained for assessment as well as the PD-L1 expression cutoff to define positivity [24]. Munari and colleagues have demonstrated that four and three core biopsy specimens are necessary to achieve an AUC with a sensitivity higher than 0.9 at the cutoff of 1% and 50%, respectively [24]. Using the same bivariate categorization (negative *vs.* positive) to Munari’s study, the concordance rate of PD-L1 expression in our study was 68% (11/16), 75% (12/16), and 87.5% (14/16) at the cutoff of 1%, 20%, and 50%, respectively, which are still too low for clinical use. Therefore, it is necessary to optimize the number of core biopsy specimens in the future study. TMB was rarely compared between small biopsy and surgical specimens in previous studies. Recently, Francesco et al. have demonstrated the technical feasibility to assess TMB in 6 out of 8 small cell blocks [25]. In the current study, we found no significant difference of this parameter between the paired samples (Fig. 4A), suggesting the feasibility to use biopsy sample as a surrogate of TMB measurement. In lung cancer, the presence of MSI was reported to be about 1% [2, 26], and was associated with higher proliferative activity [26]. Given the small sample size of our study, we cannot conclude the concordance of MSI status between biopsy and surgical specimens, although it was 100% matched in all paired samples.

Insufficient tumor tissue for molecular profiling represented a problem in advanced-stage NSCLC that accounts for about 30% of advanced-stage NSCLC patients. Besides CNB and FFPE samples, cytological samples (including conventional smears and cell blocks [CB]) derived from effusion or fine-needle aspiration biopsies are available for morphological and molecular analysis [27–29]. A recent study reported by Pepe et a. has demonstrated that TMB can be successfully analyzed on CB specimens in NSCLC patients [25]. These findings suggest that a subset of advanced-stage NSCLC patients who had insufficient tumor tissues might benefit from ICIs based on TMB analysis from CBs. Of note, the clinical utility of CBs for TMB analysis in patients with advanced stage NSCLC should be investigated in a prospective and large cohort study.

In this study, gene rearrangements were identified in two patients, P02 and P03. P02 harbored *CD74-ROS1* fusion both in the biopsy and surgical samples. P03 harbored *KDM5A-CDHR5* and concurrent *C20orf26-PPP2R2A* fusions both in the biopsy and surgical samples. DNA-seq enables the detection of novel rearrangements, while it fails to provide information on the effective transcripts of chimeric fusion. Several previous studies have reported a false negative rate of 10-15% in DNA-seq driver-negative lung adenocarcinoma patients when RNA-seq was used as a reference [30, 31]. Further RNA-seq testing or real-time polymerase chain reaction could be performed to validate the presence of DNA fusions. Moreover, DNA-seq combined with RNA-seq enables physicians to depict a more clear-cut picture of molecular features of NSCLC patients and enables more patients to benefit from efficacious treatment. We recognize the small sample size as the largest limitation of this study. However, there is currently a lack of side-by-side comparison using paired lung cancer specimens in the literature. In this regard, our work was intended to be a pilot study with the results to be taken as descriptive only. For the best interest of our participants, we adopted intraoperative puncture as the sampling method. The concurrently obtained surgical and biopsy specimens also help to eliminate chronological heterogeneity and treatment-related confounding factors, especially in locally advanced patients who require neoadjuvant therapies. Additional study using transthoracic CNB was necessary to further confirm the results. Additionally, the concordance of PD-L1 and MSI between CNB and surgical specimens cannot reach conclusive results due to problems in study design and sample size. Using at least two another independent core samples for IHC stain should be considered in the future studies. Moreover, a larger cohort of NSCLC patients who have available resection and matched CNB samples is needed to investigate the accuracy of CNB samples in detecting genomic mutations when mutations detected in resection specimens are used as references. The sensitivity, specificity, accuracy, positive predictive value, and negative predictive value for genomic mutations should be investigated in the future studies.

## Conclusions

The preliminary result of this study demonstrated that small biopsy samples obtained by CNB were the potential surrogate for NGS molecular profiling in patients with lung cancers. The snapshot of overall genetic alternations, driver mutations, and TMB captured in CNB reached a satisfactory accuracy when compared to the paired surgical specimens. NGS results yielding from CNB samples should be deemed reliable to provide instructive information for the treatment of advanced lung cancers.

## Supplementary Information

**Additional file 1: Figure S1.** Proportions of different types of alteration in resection (A) and biopsy (B) specimens. CN_amp: copy number amplification; InDel: small insertion and deletion; LGR: large genomic rearrangement.

**Additional file 2: Figure S2.** Comparative analyses of the sequencing depth (A) and allele frequency (B) between the matched alterations and specific alterations.

**Additional file 3: Figure S3. **The difference (A) and correlation (B) between resection specimen-based TMB and CNB-based TMB. RS: resection specimen; CNB: core needle biopsy; TMB: tumor mutation burden.

**Additional file 4.**

## Data Availability

All sequencing data can be viewed in NODE (http://www.biosino.org/node) by searching the project ID (OEP002199) or through the URL: http://www.biosino.org/node/project/detail/OEP002199.

## Abbreviations

NGS: Next-generation sequencing
TMB: Tumor mutational burden
MSI: Microsatellite instability
ICI: Immune checkpoint inhibitor
PD-L1: Programmed death ligand 1
PD-1: Programmed cell death protein 1
CNB: Core needle biopsy
FNA: Fine needle aspiration
PET-CT: Positron emission tomography-computed tomography
FFPE: Formalin solution and embedded in paraffin
CLIA: Clinical laboratory improvement amendments
TPS: Tumor proportion score
AF: Allele frequency
