# Supplementary material for: Accuracy of next-generation sequencing for molecular profiling of small specimen of lung cancer: a prospective pilot study of side-by-side comparison
Source: Diagn Pathol. 2022 Oct 12;17:78. doi: 10.1186/s13000-022-01255-y (PMC9554964; doi:10.1186/s13000-022-01255-y)
Supplement: Supplementary file 1 — Additional file 1: Figure S1. Proportions of different types of alteration in resection (A) and biopsy (B) specimens. CN_amp: copy number amplification; InDel: small insertion and deletion; LGR: large genomic rearrangement. [file 13000_2022_1255_MOESM1_ESM.docx]

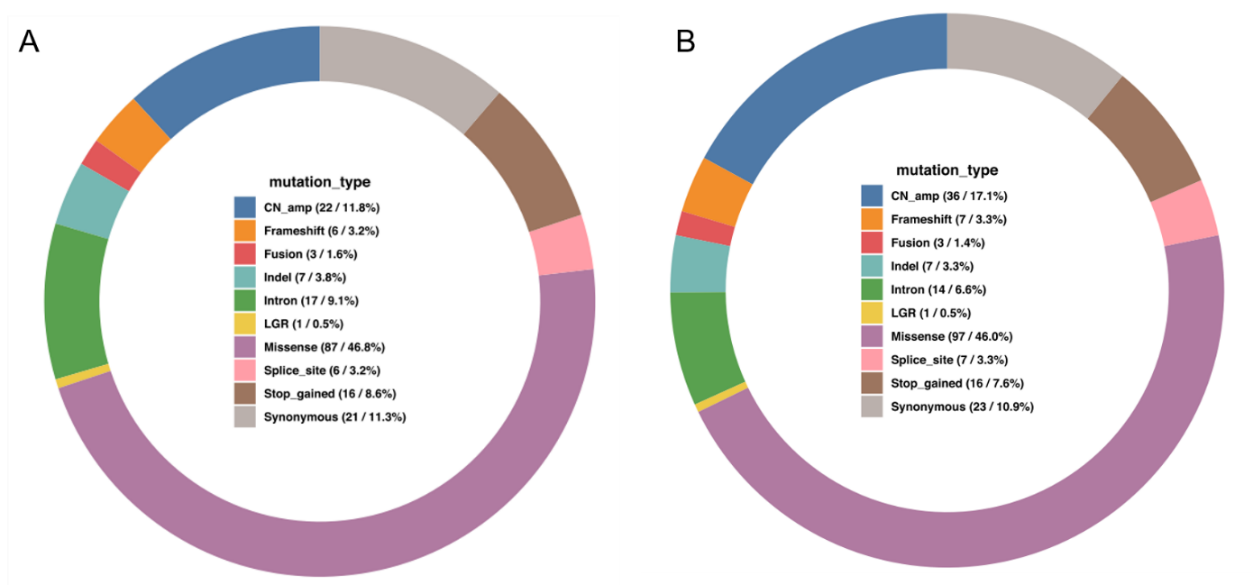


**Supplementary Figure S1.** Proportions of different types of alteration in resection (A) and biopsy (B) specimens. CN_amp: copy number amplification; InDel: small insertion and deletion; LGR: large genomic rearrangement.
