# Supplementary material for: Accuracy of next-generation sequencing for molecular profiling of small specimen of lung cancer: a prospective pilot study of side-by-side comparison
Source: Diagn Pathol. 2022 Oct 12;17:78. doi: 10.1186/s13000-022-01255-y (PMC9554964; doi:10.1186/s13000-022-01255-y)
Supplement: Supplementary file 2 — Additional file 2: Figure S2. Comparative analyses of the sequencing depth (A) and allele frequency (B) between the matched alterations and specific alterations. [file 13000_2022_1255_MOESM2_ESM.docx]

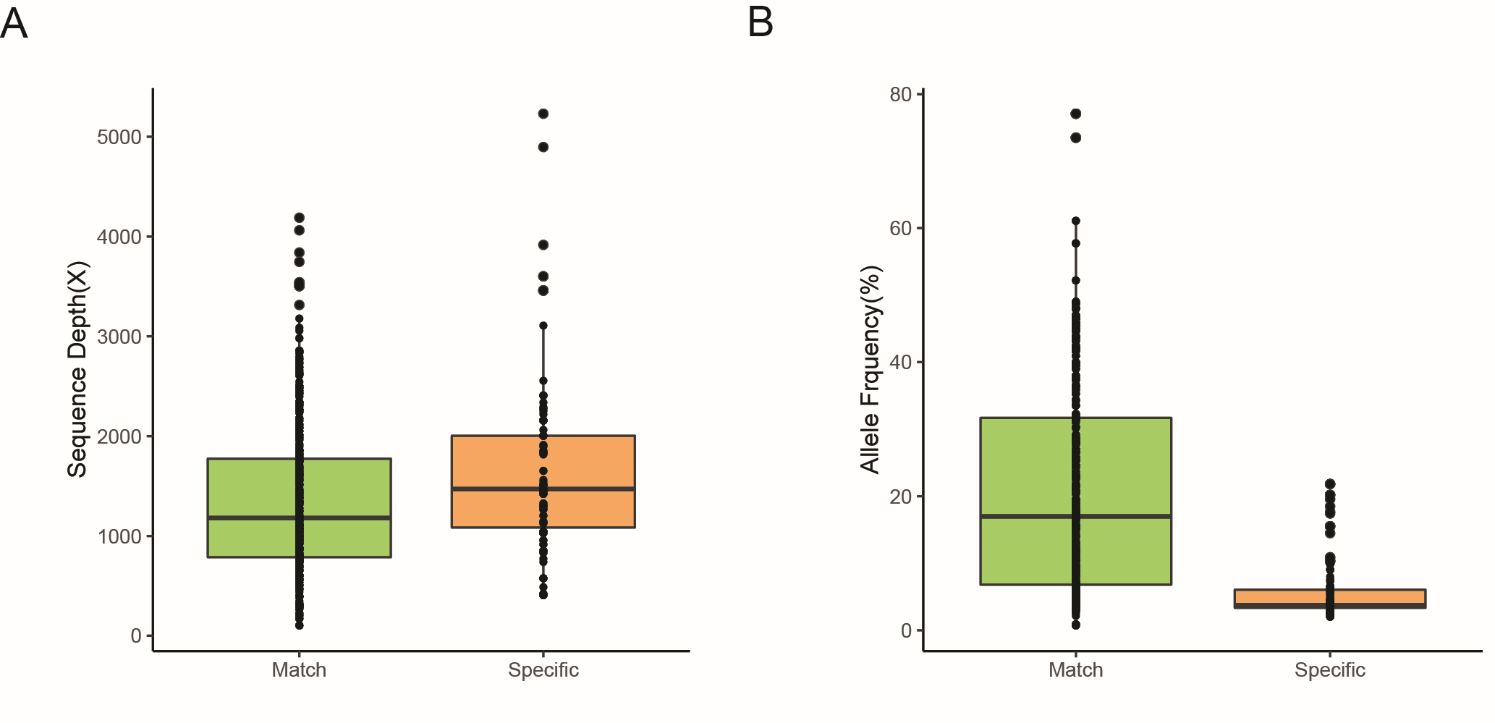


**Supplementary Figure S2.** Comparative analyses of the sequencing depth (A) and allele frequency (B) between the matched alterations and specific alterations.
