# Supplementary material for: Accuracy of next-generation sequencing for molecular profiling of small specimen of lung cancer: a prospective pilot study of side-by-side comparison
Source: Diagn Pathol. 2022 Oct 12;17:78. doi: 10.1186/s13000-022-01255-y (PMC9554964; doi:10.1186/s13000-022-01255-y)
Supplement: Supplementary file 3 — Additional file 3: Figure S3. The difference (A) and correlation (B) between resection specimen-based TMB and CNB-based TMB. RS: resection specimen; CNB: core needle biopsy; TMB: tumor mutation burden. [file 13000_2022_1255_MOESM3_ESM.docx]

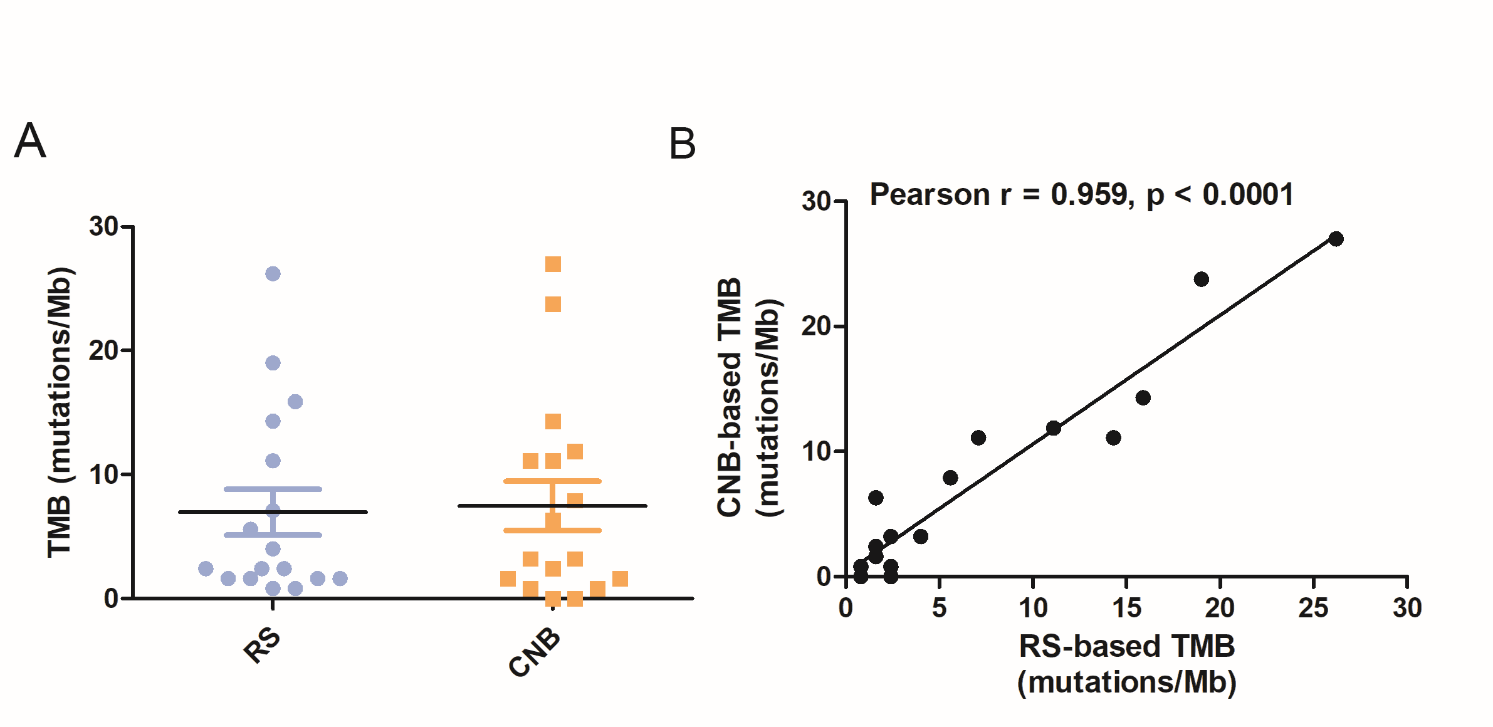


**Supplementary Figure S3.** The difference (A) and correlation (B) between resection specimen-based TMB and CNB-based TMB. RS: resection specimen; CNB: core needle biopsy; TMB: tumor mutation burden.
